# Genetic transformation of western clover (*Trifolium occidentale* D. E. Coombe.) as a model for functional genomics and transgene introgression in clonal pasture legume species

**DOI:** 10.1186/1746-4811-9-25

**Published:** 2013-07-10

**Authors:** Kim A Richardson, Dorothy A Maher, Chris S Jones, Greg Bryan

**Affiliations:** 1 AgResearch Ltd, Grasslands Research Centre, Private Bag 11008, Palmerston North 4442, New Zealand; 2 Pastoral Genomics, c/o Grasslands Research Centre, Private Bag 11008, Palmerston North 4442, New Zealand

**Keywords:** *Agrobacterium*-mediated transformation, *Trifolium occidentale*, Genetic transformation, Organogenic regeneration, Accession

## Abstract

**Background:**

Western clover (*Trifolium occidentale*) is a perennial herb with characteristics compatible for its development as an attractive model species for genomics studies relating to the forage legume, white clover (*Trifolium repens*). Its characteristics such as a small diploid genome, self-fertility and ancestral contribution of one of the genomes of *T*. *repens*, facilitates its use as a model for genetic analysis of plants transformed with legume or novel genes.

**Results:**

In this study, a reproducible transformation protocol was established following screening of *T*. *occidentale* accessions originating from England, Ireland, France, Spain and Portugal. The protocol is based upon infection of cotyledonary explants dissected from mature seed with the *Agrobacterium tumefaciens* strain GV3101 carrying vectors which contain the *bar* selection marker gene. Transformation frequencies of up to 7.5% were achieved in 9 of the 17 accessions tested. Transformed plants were verified by PCR and expression of the *gusA* reporter gene, while integration of the T-DNA was confirmed by Southern blot hybridisation and segregation of progeny in the T_1_ generation.

**Conclusions:**

Development of this protocol provides a valuable contribution toward establishing *T*. *occidentale* as a model species for white clover. This presents opportunities for further improvement in white clover through the application of biotechnology.

## Background

**Figure 1:**
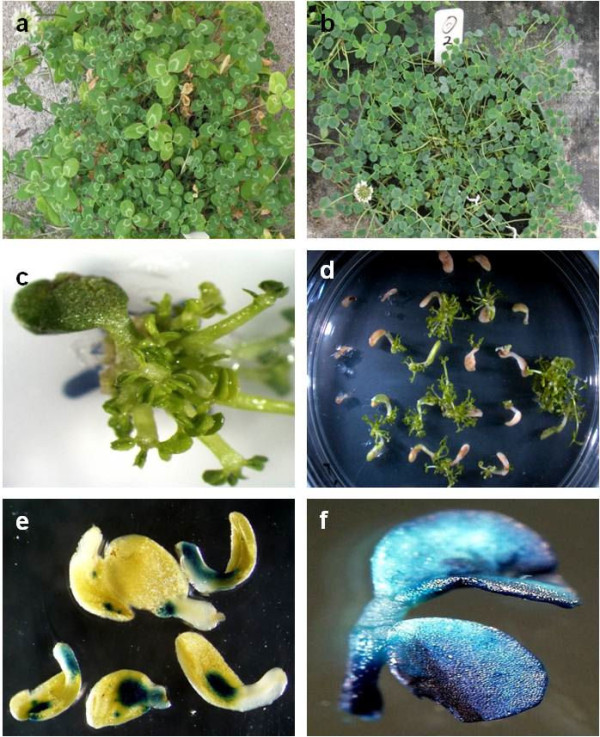
**Western clover (*T. occidentale*), shoot regeneration and transformation. (a)** white clover (*T*. *repens*); compared to **(b)***T*. *occidentale***(c)** prolific regeneration of shoots from an AZ4270 cotyledon following three weeks culture on 1C; **(d)** shoot regeneration under ammonium glufosinate selection following *Agrobacterium*-mediated transformation of cotyledon explants; **(e)** transient GUS expression on cotyledonary explants three days after inoculation with Agrobacterium broth; **(f)** GUS activity in a trifoliate leaf excised from stably transformed plants approximately eight weeks after transformation.

White clover (*Trifolium repens* L., Figure 1a) is the most extensively used perennial legume in temperate grazed pastoral systems grown for its nutritional value and ability to fix atmospheric nitrogen [1,2]. This stoloniferous, clonal herb [3] originating from the grasslands of Europe, Western Asia and North Africa has an allotetraploid (2n = 4x = 32) genome derived from two diploid ancestors *T*. *pallescens* (2n = 2x = 16) and *T*. *occidentale* (2n = 2x = 16) [4]. The conventional breeding techniques used for plant improvement have resulted in the release of many commercial cultivars, however, it is now recognised that biotechnology approaches hold the promise of additional genetic gain in this species through the introduction of novel traits [5].

Expression of heterologous genes in white clover has identified the potential for crop improvement through biotechnology including the accumulation of fructan as a storage carbohydrate [6], increased accumulation of sulphur rich amino acids in foliar tissues [7,8], insect pest resistance through plants expressing a modified cry1Ba [9], Alfalfa Mosaic Virus resistance in field trial experiments [10] and the investigation of white clover as a delivery mechanism for edible vaccines [11].

In recent years significant genomic resources both for white clover and model legume species have become available [12-14]. While transformation protocols for white clover are readily available [15-18]; the ability to exploit these resources is limited by a genetic composition resulting in self incompatiblility, cross pollination, allotetraploidy and amphidiploid inheritance with high levels of heterozygosity making the application of genomic technologies problematic at best [19]. Such characteristics are complicating factors for the genetic analysis of transgene inheritance in white clover and are an issue which is common to many crop species. Whereas elite cultivars are generally developed from multiple parents and therefore represent a heterogeneous mixture of heterozygous individuals, the inter-crossing between closely related genotypes – as required for the development of transgenic progenies - often leads to a loss of heterozygosity and inbreeding depression [20,21].

To overcome this problem, western clover (*T*. *occidentale*, Figure 1b) appeals as a model for gene expression and determination of gene function in white clover. *T*. *occidentale* is diploid, possesses a small genome (approximately 0.5 pg/1C) of similar size to *Medicago truncatula* and *Lotus japonicas*[22] and is a proposed progenitor of *T*. *repens*, therefore, most genes will be highly related. It has both self-fertile and self-incompatible accessions, a relatively short generation time and is able to fix nitrogen in symbiotic association with *Rhizobium* bacteria [19]. Its natural habitat is along coastal regions of Europe and the British Isles exposed to the Gulf Stream and until its description as a new species was regarded as a variety of white clover [23]. Tools such as the establishment of EMS mutants [19] and a genome which is currently being sequenced [24] make *T*. *occidentale* an ideal candidate as a model species for functional genomics in pasture legumes.

Ploidy manipulation by *in vitro* treatment with colchicine has been used in kiwifruit and peanut as a breeding strategy to generate hybrids between closely related diploid and tetraploid species [25,26]. When applied to *T*. *occidentale* this strategy allows the hybridisation of *T*. *occidentale* transgenics directly with white clover. This hybridisation approach following characterisation of the transgene in the *T*. *occidentale* model system provides a viable strategy for the rapid transfer of new traits into commercially relevant germplasm. In addition, reports of the generation of a synthetic white clover through the hybridisation of *T*. *occidentale* with *T*. *pallescens*[4] and a novel hybrid from *T*. *occidentale* with *T*. *nigrescens*[27] offer exciting possibilities for the introgression of GM traits into a range of novel clover germplasm. The use of *T*. *occidentale* as a genetic bridge to other *Trifolium* species provides significant advantages for GM breeding in forage legumes.

Here, we report on the transformation capacity of *T*. *occidentale* accessions collected from England, Ireland, France, Portugal and Spain. In these experiments we: (1) determined optimal conditions for organogenic regeneration of an English accession, (2) assessed regeneration in accessions from England, Ireland and France, (3) performed *Agrobacterium*-mediated transformation experiments with seventeen accessions to regenerate transformed plants expressing a *gusA* reporter gene, (4) characterised the plants by Southern blot hybridisation and PCR analysis to demonstrate stable integration of the T-DNA, and (5) examined the segregation of the transgene in T_1_ progeny.

To our knowledge this is the first description of a complete protocol for the reliable production of transformed *T*. *occidentale*. As with white clover; the system utilizes cotyledons dissected from mature seed as an explant for plant regeneration. Rapid regeneration was achieved via direct organogenesis and plants were recovered from a number of the accessions tested.

## Results and discussion

### Shoot regeneration

Primary requirements for plant transformation include the availability of target tissues competent for plant regeneration at high frequency, a method to introduce genetic constructs into regenerable cells and a procedure to select and regenerate plants at a satisfactory frequency [28]. Based upon our experience with white clover, we tested the ability of cotyledonary explants to regenerate on a range of culture media containing different plant growth regulator (PGR) combinations. Accession AZ4270 was used in the first phase of this process to identify an optimal growth medium for the regeneration of plants from dissected cotyledons.

PGR treatment combinations comprising cytokinin BA or TDZ and auxin IBA or NAA were tested. Each combination was developed around PGR regimes cited in successful legume tissue culture protocols. These included those described for *T*. *repens *[16,29], *Medicago truncatula *[30,31] and *Lotus japonicus *[32,33]. PGR treatment combinations comprised; (1) BA and IBA, (2) BA and NAA, (3) TDZ and IBA and (4) TDZ and NAA at varying concentrations. The experiment used 80 cotyledons per treatment (four replicate plates of 20 explants per plate) for each of the 42 different media.

**Figure 2:**
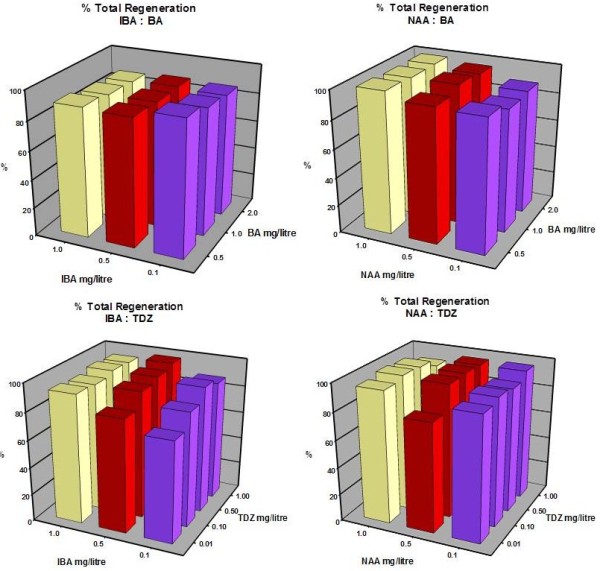
**Influence of PGR regime on the frequency of shoot regeneration from cotyledonary explants of AZ4270.** Each data point represents the mean of 4 replicate plates containing 20 explants per plate.

Explants were cultured on plant growth media for four weeks without subculture. Regeneration frequencies (number of explants regenerating per plate) were assessed at this time (Figure 2, Additional file 1: Table S1). A relatively uniform production of plantlets with high levels of regeneration (86-94%) was encountered over the range of PGR combinations and concentrations; however, there were clear visual differences in the fitness and average number of shoots produced per explant. Therefore, we also examined the vigor, hyperhydricity and average number of shoots regenerating per explant in each treatment (data not shown). Treatments resulting in the regeneration of greater than five, morphologically normal, shoots per explant (Figure 1c) were an important parameter in this assessment.

Shoots regenerating in Treatment 1 were more robust in comparison to those from Treatments 2, 3 or 4. Hyperhydricity was encountered at slightly higher levels in Treatment 2 compared to Treatment 1 and at unacceptably high levels in Treatments 3 and 4 where TDZ was utilized as the source of cytokinin. This is in contrast with Ding *et al*. [15] who report high levels of plant regeneration in five *Trifolium* species using TDZ, although the phenomena of hyperhydricity induced by TDZ has been reported for other plant species [34-36].

**Figure 3:**
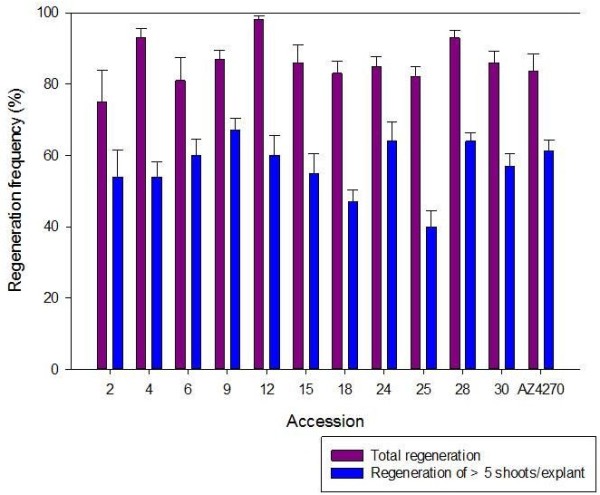
**Regeneration frequency of eleven accessions compared to AZ4270 on medium 1C.** Data were scored following four weeks of culture.

Medium C from Treatment 1 (1C, 0.5 mgl^-1^ BA, 1.0 mgl^-1^ IBA) resulted in superior regeneration of *T*. *occidentale* shoots. To determine the utility of 1C across a range of germplasm, we tested a further 11 accessions originating from France (5), Ireland (4) and England (2) for regeneration capability. Frequencies of 75 to 98% were achieved indicating a genotype dependent response but confirming 1C as a suitable regeneration medium for this species (Figure 3). Based on these assessments of regeneration efficiency and prolific shoot formation, Medium 1C was chosen to establish a transformation system for *T*. *occidentale*.

Shoots recovered from each of the treatments were assessed for their ability to develop roots, under *in vitro* conditions following culture, on a growth regulator-free medium containing half strength MS salts and 3% maltose. A total of 21 shoots were tested and root formation assessed after five weeks of culture. Root formation of > 50% was achieved in shoots from each of the media tested. Rooted plants were then transferred to the greenhouse and observed to be morphologically similar to greenhouse grown seedlings of AZ4270.

A shoot regeneration frequency of over 95% was similar to that reported for white clover on the same plant growth regulator regime at comparable concentrations [29]. Therefore, it appears that *T*. *occidentale* behaves in much the same way as *T*. *repens* and upwards of five shoots per explant can be expected on most media. However, using different PGRs we have found that a combination of BA and IBA is optimal for production of normal shoots at a high regeneration frequency. TDZ has often been shown to be more successful than BA for the regeneration of shoots in many plant species including some *Trifolium* species [15,37] but this was not observed here and resulted in poor quality material that was highly hyperhydric.

#### *Agrobacterium*-mediated transformation

For *T*. *occidentale* to be a good candidate for use as a genetic model it is important that it can be easily transformed and whole plants regenerated. As legume transformation is often genotype dependent [38], a series of experiments were carried out to compare the response of 16 accessions to *Agrobacterium*-mediated plant transformation. These accessions, collected from 4 sites in Portugal and 12 sites in Spain, represented a range of germplasm, both self-fertile and self-incompatible, showing variability in vigour and growth habit, with several displaying leaf mark characteristics similar to those of *T*. *repens*.

Based on the efficient regeneration achieved from AZ4270, Medium 1C was selected for use in the co-cultivation and regeneration/selection phases of the transformation experiments. For comparison we also tested Medium 2D (0.5 mgl^-1^ BA, 0.1 mgl^-1^ NAA) which is used for white clover regeneration in our laboratory. Transformation was based upon protocols described for other *Trifolium* species [15-17] by co-cultivation of cotyledons dissected from mature seed with the *Agrobacterium tumefaciens* strain GV3101 harbouring the binary vector pHZBar-intGUS (Additional file 2: Figure S1) carrying the *bar* selection marker and the *gusA* reporter gene. Shoots regenerated directly from the cotyledonary axil via organogenesis in a similar manner to that described for white clover [29]. Putatively transformed plants were recovered from explants 4 to 6 weeks post transformation (Figure 1d).

Bacterial contamination was difficult to control in several accessions 34, 36, 39, 43, 49 and 53 where growth of explants was inhibited. These lines were discarded from further transformation assessments. This contamination appeared to arise from endogenous bacteria inherently associated with the testa of imbibed seeds and was often uncontrollable even with bacteriostatic compounds such as timentin or cefotaxime. These endophytic bacteria can be difficult to eliminate by simple surface sterilization [39,40], however, such high levels of contamination are rarely encountered in commercially produced white clover seed. This high bacterial load was not unexpected as the seed used in these experiments were only one generation removed from wild populations. It is anticipated that successive seed collections in a phytosanitary environment will provide a clean source of material for transformation.

Activity of an intron containing *gusA* reporter gene was used to monitor transfer of T-DNAs into cells of the explant. Transient expression was observed in approximately 65% of explants, following 3 days of co-cultivation (Figure 1e) indicating that the transformation parameters routinely used for white clover were also likely to be suitable for gene transfer into *T*. *occidentale*. GUS activity was also used to identify stably transformed plants (Figure 1f) and to determine the transformation frequency of accessions which ranged from 0 to 7.5 transformed plants per one hundred explants (Table 1).

**Table 1:** **Summary of transformation frequencies obtained for *T***. ***occidentale *accessions**

**Accession**	**Media**	**Total no. explants**	**Total no. plants**	**Transformation frequency**
AZ4270	1C	250	1	0.40
	2D	250	0	0.00
32	1C	260	0	0.00
	2D	260	0	0.00
37	1C	180	0	0.00
	2D	180	3	1.67
38	1C	240	0	0.00
	2D	240	0	0.00
41	1C	150	4	2.67
	2D	150	2	1.33
44	1C	180	4	2.22
	2D	180	1	0.56
45	1C	220	12	5.45
	2D	220	12	5.45
48	1C	200	5	2.50
	2D	200	15	7.50
52	1C	180	7	3.89
	2D	180	4	2.22
54	1C	200	0	0.00
	2D	180	2	1.11
59	1C	220	2	0.91
	2D	150	1	0.67

The data are the means of two independent experiments.

Transformed plants were recovered from 9 of the 17 accessions tested with transformation frequencies obtained similar to those reported previously for white clover, red clover and subterranean clover [15]. The variable recovery of transformed plants across accessions and between the two media reflects the genotype dependent nature of transformation which is commonly encountered in many *Trifolium* species. For example, in AZ4270, a transformation frequency of 0.4% was obtained on medium 1C while no plants were recovered on medium 2D. Similarly, no plants were recovered from accessions 32 or 38 regenerating on either media. Accessions 37, 41, 44, 52, 54 and 59 gave transformation frequencies in the range of that obtainable from AZ4270 or *T*. *repens*. Frequencies for accessions 45 and 48 were slightly higher with an average frequency of 5.4% achieved for 45 on both media whereas plants were recovered at a frequency of 2.5% on 1C and 7.5% on 2D for genotype 48. Accession 41 was the best performing self-fertile accession with a transformation frequency of 2.7% on the 1C medium.

### Characterisation of transgenic plants

Primary transformant (T_0_) plants were assayed for integration of the T-DNA into the genome and expression of the transgene. Histochemical GUS assays and PCR, performed on leaf pieces from putatively transformed plants, were used to confirm the transgenic status of plants. Results of plants staining positive for GUS activity or by PCR analysis were combined to determine the transformation frequency.

**Figure 4:**
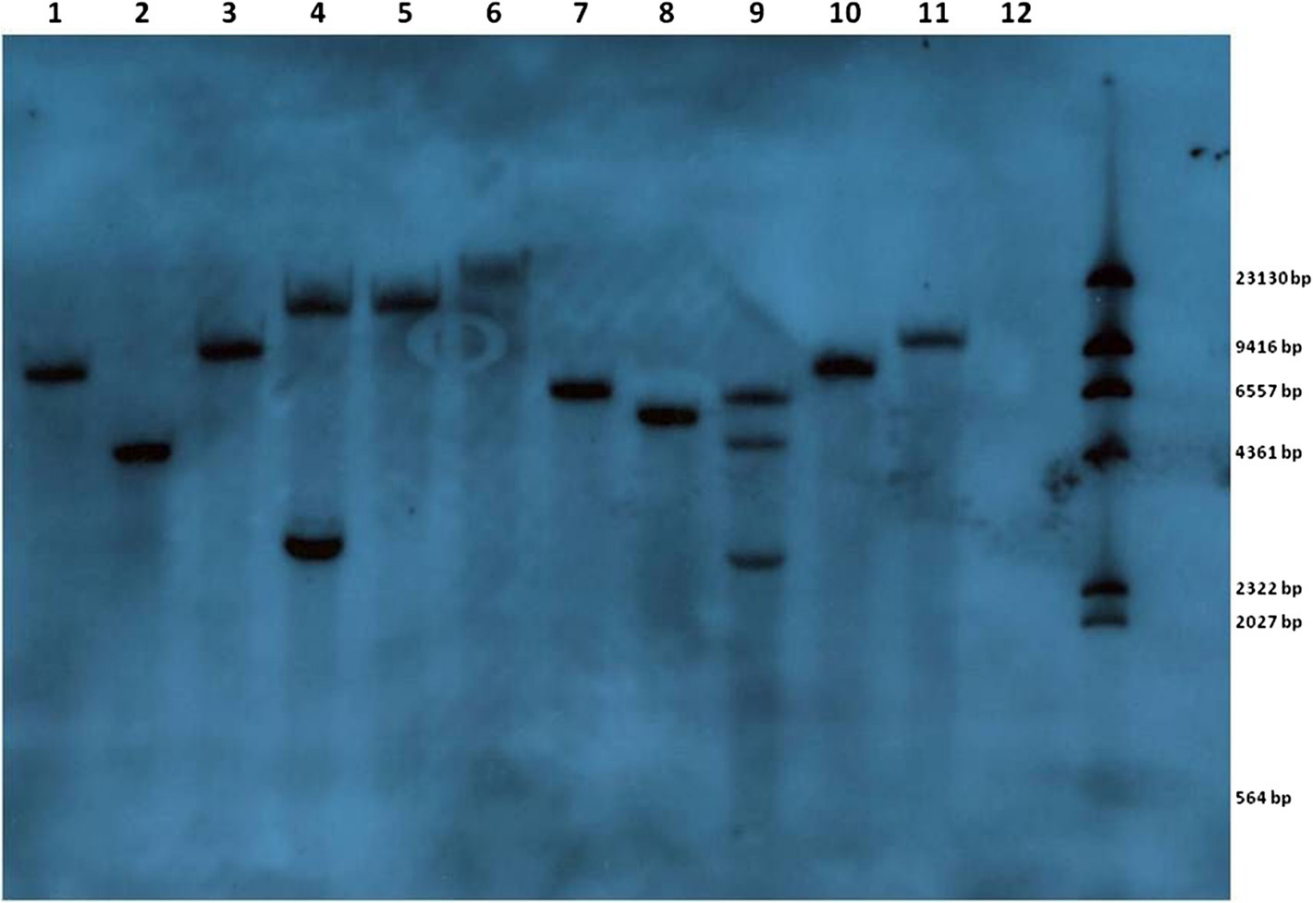
**Southern blot hybridisation of *T. occidentale *T**_**0 **_**translines.** Total genomic DNA was prepared from foliar tissues of independently transformed plants and a non-transformed wild type control. DNAs were digested with the restriction enzyme *Eco*RV and probed with a DIG-labelled fragment of the 3′ region of the *gusA* reporter gene. Lanes 1–11 transformed plants, lane 12 non-transformed control and lane 13 is the DIG labelled DNA Molecular Weight Marker II (Roche).

Southern blot hybridization was used to estimate the number of T-DNA insertions, or copy number, per line. Genomic DNA was digested using *Eco*RV and hybridized using a non-radioactive DIG probe designed to bind to the 3’ region of the *gusA* reporter gene situated between the *Eco*RV site and the Right Border of the T-DNA. This resulted in bands of greater than 2.8 KB. An example of the hybridization patterns is shown in Figure 4. The different integration patterns indicate independent transformation events were generated. Between one and three T-DNA integrations per line were identified with 8 of the 11 lines tested showing a single insertion of the T-DNA.

### Segregation of T_1_ progeny

The hemizygous nature of the T-DNA insert means that characterization of transgene expression and phenotype can be problematic in the T_0_ and subsequent generations of an obligate out crossing and highly heterozygous plant species such as white clover. The tetraploid genome of white clover may further complicate expression patterns. In a self-fertile diploid species it is a relatively simple process to generate genetically identical lines for analysis with plants which are homozygous for the transgene. However, in an out crossing species this generally requires cross pollination between closely related genotypes over at least two generations and is difficult to achieve without the introduction of inbreeding depression due to a loss of heterozygosity.

To overcome this difficulty the concept of “isogenic transformation” in white clover has been demonstrated as a means by which transformed and non-transformed plants may be compared in the same genetic background [41]. The method involves (i) transformation and (ii) culture, respectively, of cotyledon pairs dissected from individual seeds with the resultant plant pairs being used for a comparative analysis. But this is a rather labour intensive, time consuming approach requiring the maintenance of large numbers of non-transformed partners prior to the selection, regeneration and identification of transformed plants. Furthermore, despite being isogenic, the resulting T_0_ plants are hemizygous and a complicated crossing procedure is still required to establish populations which possess a homozygous transgene. Here we show that the transformation capacity of self-fertile accessions of *T*. *occidentale* provides a viable alternative to isogenic transformation. Transformed lines of interest in the T_0_ generation may be identified and T_1_ seed populations harvested from these within 5–7 weeks following flowering.

Transmission of the transgene was demonstrated in 4 independently transformed lines derived from the self-fertile accession 41 transformed with a construct designed to co-express the Diacylglycerol acyltransferase 1 gene from nasturtium and a modified oleosin gene from sesame [42]. T_0_ plants were grown to maturity, self-pollinated to produce seed and progeny grown to produce T_1_ plants in order to confirm inheritance of the transgene in the next generation. Progeny from three lines containing a single T-DNA insert and one line containing three T-DNA insertions (as determined by Southern blot hybridization) were scored for transmission of the transgene by PCR (Table 2). The expected Mendelian inheritance, a 3:1 segregation ratio, was observed in 2 of the single copy T-DNA lines (AAT6501 and AAT6503). The third single copy T-DNA line AAT6502 did not conform to Mendelian expectations and segregated in a 2:1 ratio (Chi squared = 1.24) which is indicative of a T-DNA insertion causing a lethal knockout of homozygous progeny. The 3 copy T-DNA line (AAT6505) also segregated with a 3:1 ratio (Chi squared = 0.28) suggesting the insertion of all T-DNA’s at a single, or closely linked, loci.

**Table 2:** **Segregation of the *bar *selection marker in T**_**1 **_**progeny of *T***. ***occidentale *translines**

**Plant line**	**Total no. seedlings**	**Observed**		**Expected**		**Chi-square**	**P value**
		PCR+	PCR-	PCR+	PCR-		
AAT6501	53	44	9	40	13	1.63	0.202
AAT6502	58	35	23	44	14	7.63	0.006
AAT6503	56	43	13	42	14	0.10	0.758
AAT6505	76	59	17	75	1	19	2.3×10^-58^

Presence of the *bar* gene was determined by PCR assay of genomic DNA isolated from leaf tissues of T_1_ seedlings. For AAT6501, AAT6502 and AAT6503 the expected ratio is based upon a 3:1 segregation of a single copy T-DNA at a single insertion locus and a 63:1 segregation for AAT6505 of three T-DNA’s at separate insertion loci.

Seed was recovered from a further three independently transformed lines suggesting that fertility and seed set is not adversely affected in mature plants following transformation. Yields of 130 to several thousand seed per plant were achieved. The phenotype of all primary transformants and progenies appeared similar to non-transformed control plants when grown in a contained greenhouse environment.

## Conclusions

Efficient genetic transformation in nine accessions of *T*. *occidentale* originating from England, Portugal and Spain has been demonstrated. An optimal media has been identified for use in a protocol adapted from Voisey *et al*. 1994 [17] and the transformation frequencies obtained were comparable to those routinely observed for *T*. *repens* in our hands. Based upon the frequency of shoot regeneration achieved for the fully self-fertile accessions from England, Ireland and France it is likely that the recovery of transformed plants is possible across a wider range of germplasm than tested in these experiments. Stable inheritance of the transgene, its segregation in a Mendelian fashion and reliable seed generation was demonstrated in *T*. *occidentale*. These characteristics when coupled with an ability to hybridise with *T*. *palescens* (and other diploid clovers) offers the possibility of a viable method for the use of *T*. *occidentale* as a “genetic bridge” for the introgression of novel traits into white clover.

Development of this protocol is a significant contribution toward establishing *T*. *occidentale* as a genetic model allowing the study of agriculturally or biologically important questions in white clover. Its diploid genome, self-fertility and close ancestral relationship to *T*. *repens* combined with the availability of genomic resources underlie its potential as a model for testing gene function in *Trifolium* species.

## Materials and Methods

### Plant material

Seed of 28 accessions of *T*. *occidentale* (Table 3) originating from England, Ireland, France, Portugal and Spain were obtained from the Margot Forde Germplasm Centre at AgResearch. Seed of accession AZ4270 (England) was used for both shoot regeneration and transformation experiments whereas all other accessions were assessed either for regeneration or capability in *Agrobacterium*-mediated transformation. Aliquots of up to 100 seeds were pre-treated by soaking in concentrated sulphuric acid for ten minutes prior to surface sterilization. This acid scarification allows seed to subsequently imbibe in preparation for germination [43]. Acid was thoroughly washed from seed by rinsing with water and then seeds were surface sterilized by soaking in bleach (5% available chlorine) for approximately 10 minutes followed by up to 6 washes in sterile distilled water. The seeds were then allowed to imbibe overnight at room temperature or for up to 3 days at 4°C. Seeds were dissected under a binocular microscope as described by White and Voisey [29] to separate the imbibed cotyledons. Excised cotyledons were placed adaxial side uppermost on the plant growth media.

**Table 3:** ***T***. ***occidentale *accessions used in this study**

**Accession**	**Collection site**	**Location**	Fertility*	**Use in this study**
AZ4270	Cornwall	England	SF	Shoot regeneration/ Transformation
2	Skerries	Ireland	SF	Shoot regeneration
4	Bull Island	Ireland	SF	Shoot regeneration
6	Kilmore Quay	Ireland	SF	Shoot regeneration
9	St Helens	Ireland	SF	Shoot regeneration
12	Mayon Cliff	England	SF	Shoot regeneration
15	Mullion Cliff (Cornwall)	England	SF	Shoot regeneration
18	Le Guilvinec	France	SF	Shoot regeneration
24	Lampaul-Plouarzel (Brittany)	France	SF	Shoot regeneration
25	Point du Corson (Brittany)	France	SF	Shoot regeneration
28	Dunes St Marguerite (Brittany)	France	SF	Shoot regeneration
30	Flamanville (Normandy)	France	SF	Shoot regeneration
32	Leca da Palmeira	Portugal	SF	Transformation
34	Villa Cha	Portugal	SF	Transformation
36	Costelo do neiva	Portugal	SF	Transformation
37	Carreco	Portugal	SF	Transformation
38	Saxian	Spain	SF	Transformation
39	Cabo Silleiro (Baredo)	Spain	SF	Transformation
41	Cabo de Corrubedo lighthouse	Spain	SF	Transformation
43	Punta louro lighthouse	Spain	SF	Transformation
44	Praia de Larino	Spain	SI	Transformation
45	Faro de Finisterra	Spain	SI	Transformation
48	Faro de Cabo village	Spain	SI	Transformation
49	Camarinas	Spain	SI	Transformation
52	Beo Peninsula	Spain	SI	Transformation
53	Punta Frouxeira lighthouse	Spain	SI	Transformation
54	Punta Frouxeira beach	Spain	SI	Transformation
59	Playa de San Antolin	Spain	SF	Transformation

*SI and SF indicate self-incompatible or self-fertile respectively.

### Plant growth media and shoot regeneration

The basal medium for all *in vitro* culture contained Murashige and Skoog (MS) salts [44] and B5 vitamins [45] supplemented with 30 gl^-1^ maltose and 8 gl^-1^ phytoagar (Gibco, BRL) and the combinations of PGRs as described below. Treatments containing PGRs; 6-Benzyladenine (BA), Indole-3-butyric Acid (IBA), Napthaleneacetic acid (NAA) and Thidiazuron (TDZ) were used in a media optimization experiment which comprised forty two media. These media contained combinations of the following: 0.5 to 2.0 mgl^-1^ BA and 0.1 to 1.0 mgl^-1^ IBA; 0.5 to 2.0 mgl^-1^ BA and 0.1 to 1.0 mgl^-1^ NAA; 0.01 to 1.0 mgl^-1^ TDZ and 0.1 to 1.0 mgl^-1^ IBA; 0.01 to 1.0 mgl^-1^ TDZ and 0.1 to 1.0 mgl^-1^ NAA. All media were adjusted to a pH of 5.8 with KOH before autoclaving at 121°C for 15 minutes. PGRs were filter sterilized and added after autoclaving. All experiments used 9.0 cm Petri dishes sealed with catering film and were conducted in culture rooms set to a constant temperature of 25°C with a 16 hour photoperiod using a combination of Cool White and Grolux fluorescent lamps at light intensity of 60 mol m^-2^ s^-1^.

### Bacterial strains and plasmids

The binary vector pHZBar-intGUS was used for all transformation experiments. This vector, a derivative of pART27 [46], contains the *bar* selection gene expressed from the CaMV 35S promoter and a *gusA* reporter gene also under the expression of the CaMV 35S promoter. The plasmid was transferred into *A*. *tumefaciens* strain GV3101 [47] by triparental mating [48]. Transformants were selected on YM plates containing 200 mgl^-1^ Spectinomycin and successful mobilisation of the plasmid into *Agrobacterium* was confirmed by restriction mapping following preparation of plasmid DNA from the bacterial culture. A starter culture was prepared using the method of Tingay *et al*. [49] and used to provide a standardized inoculum for transformation experiments.

#### *Agrobacterium*-mediated transformation

A culture of pHZBar-intGUS/GV3101 was added to 25 ml of Mannitol Glutamate Luria (MGL) broth containing 100 mgl^-1^ Spectinomycin. Bacterial cultures were grown overnight (16 hours) on a rotary shaker (200 rpm) at 28°C. Cultures were harvested by centrifugation (3000 × g), the supernatant removed and the cells re-suspended in a 5 ml solution of 10 mM MgSO_4_ in preparation for plant transformation. Freshly dissected cotyledons were inoculated with 3 μl of *Agrobacterium* suspension and co-cultivated for 72 hours at 25°C under a 16 hour photoperiod.

### Selection of transformed plants

Co-cultivated cotyledons were transferred to plates containing regeneration medium supplemented with 2.5 mgl^-1^ ammonium glufosinate (Fluka) and 300 mgl^-1^ Timentin (GlaxoSmithKilne) and returned to the culture room. Regenerated shoots were transferred to rooting medium (0.1 mgl^-1^ BA, 0.05 mgl^-1^ NAA) and then to hormone-free basal medium supplemented with ammonium glufosinate and Timentin for further growth before transfer to the greenhouse. Regenerating shoots were subcultured to fresh media containing selection at two weekly intervals.

### DNA extraction and PCR

PCR analysis was performed to confirm stable integration of the T-DNA into the genome for plants recovered from transformation experiments. Genomic DNA was extracted from approximately 50 mg of *in vitro* grown leaves using the Genomic DNA Mini Kit (Geneaid). Primer pairs specific to the ocs3′ polyadenylation signal (ocs3′-1f, 5′-GATATGCGAGACGCCTATGA-3′; ocs3′-1r, 5′-GAGTTCCCTTCAGTGAACGT-3′), *bar* gene (bar-3, 5′-CAGGAACCGCAGGAGTGGA-3′; bar-4, 5′-CCAGAAACCCACGTCATGCC-3′) and *gusA* reporter gene (*gusA*-1f, 5′-AACAGTTCCTGATTAACCACAAACC-3′; *gusA*-1r, 5′-GCCAGAAGTTCTTTTTCCAGTACC-3′) were used to produce amplification products of 439 bp, 372 bp and 634 bp respectively. Control reactions comprising plasmid DNA template, non-transformed plant DNA or water only were also included. The protocol for PCR reactions consisted of: an initial denaturation of 94°C for 5 minutes, 30 cycles of 95°C 30 s, 55°C 15 s, 72°C 1 min, and an extension of 72°C for 10 min. Amplification products were resolved on 1.0% agarose gels by gel electrophoresis in TAE buffer and visualized with a Bio-Rad Gel Doc imaging sytem.

### Histochemical GUS assay

GUS activity was determined in the leaves of primary transformed (T_0_) plants using a histochemical assay [50]. The reagent X-Gluc (1.0 mg/ml, pH7.0, Duchefa) was used to stain leaves of *in vitro* plants regenerated under ammonium glufosinate selection. A negative control of non-transformed leaf tissue and a positive control (transgenic tissue expressing GUS from the CaMV35S promoter) were included in the assay.

### Southern blot analysis

Genomic DNA was extracted from leaf material of greenhouse grown plants for Southern blot hybridization using the method of [51]. DNA (20 μg) was digested with *Eco*RV, separated on a 0.8% agarose gel and transferred onto a nylon membrane (Roche) using capillary transfer with 0.4N NaOH. A 634 bp probe to the *gusA* gene using primers *gusA*-1f and *gusA*-1r was prepared using the DIG PCR synthesis kit. Prehybridization (1 hour) and hybridization (12 hours) were performed at 45°C using standard buffers (Roche). Detection was achieved using a non-radioactive method according to the manufacturer’s protocol with CDP-Star as the chemiluminescent substrate. Light signals were detected on X-ray film (Roche).

## Abbreviations

BA: 6-Benzyladenine; GUS: β-Glucuronidase; IBA: Indole-3-butyric Acid; MS: Murashige and Skoog medium; NAA: Napthaleneacetic acid; PCR: Polymerase chain reaction; PGR: Plant growth regulator; TDZ: Thidiazuron; X-Gluc: 5-bromo-4-chloro-3-indoyl glucuronide.

## Competing interests

The authors declare they have no competing interests.

## Authors’ contributions

KAR, CSJ and GB designed the experiments. KAR constructed the vector pHZBar-intGUS, performed the Southern Blot Hybridisation and wrote the manuscript. DAM performed all tissue culture, genetic transformation experiments and maintained plants in the greenhouse. All authors read and approved the final manuscript.

## Supplementary Material

Additional file 1: Table S1 Regeneration frequencies from explants of AZ4270 achieved for each PGR treatment. Click here for file

Additional file 2: Figure S1 Diagram of the T-DNA of pHZBar-intGUS. Click here for file

## Research area

Model species for white clover genomics.
