# Supplementary material for: Genetic transformation of western clover (Trifolium occidentale D. E. Coombe.) as a model for functional genomics and transgene introgression in clonal pasture legume species
Source: Plant Methods. 2013 Jul 10;9:25. doi: 10.1186/1746-4811-9-25 (PMC3716983; doi:10.1186/1746-4811-9-25)
Supplement: Additional file 1: Table S1 — Regeneration frequencies from explants of AZ4270 achieved for each PGR treatment. [file 1746-4811-9-25-S1.pdf]

Table S1 Influence of PGR regime on the frequency of shoot regeneration from cotyledonary explants of AZ4270. Each data point represents the mean of 4 replicate plates containing 20 explants per plate.

PGR treatment (mgl^{-1})			% Regeneration Total (SEM)	% Regeneration >5 Shoots (SEM)
Treatment 1				
1a	BA 0.5	IBA 0.1	93.8 (2.5)	70.0 (3.5)
1b	BA 0.5	IBA 0.5	88.8 (4.3)	71.3 (7.5)
1c	BA 0.5	IBA 1.0	90 (2.0)	73.8 (7.2)
1d	BA 1.0	IBA 0.1	88.8 (3.8)	60.0 (2.0)
1e	BA 1.0	IBA 0.5	87.5 (4.8)	66.3 (5.2)
1f	BA 1.0	IBA 1.0	83.8 (4.7)	61.3 (3.2)
1g	BA 2.0	IBA 0.1	86.3 (5.2)	58.8 (3.8)
1h	BA 2.0	IBA 0.5	87.5 (2.5)	66.3 (4.3)
1i	BA 2.0	IBA 1.0	76.3 (8.0)	48.8 (8.3)
Treatment 2				
2a	BA 0.5	NAA 0.1	93.8 (3.2)	60 (3.5)
2b	BA 0.5	NAA 0.5	95 (3.5)	78.8 (3.8)
2c	BA 0.5	NAA 1.0	100 (0)	67.5 (6.3)
2d	BA 1.0	NAA 0.1	87.5 (4.8)	63.8 (5.5)
2e	BA 1.0	NAA 0.5	98.8 (1.3)	78.8 (3.2)
2f	BA 1.0	NAA 1.0	98.8 (1.3)	66.3 (8.8)
2g	BA 2.0	NAA 0.1	88.8 (3.2)	57.5 (8.5)
2h	BA 2.0	NAA 0.5	96.3 (2.4)	83.8 (6.9)
2i	BA 2.0	NAA 1.0	98.8 (1.3)	82.5 (1.4)
Treatment 3				
3a	TDZ 0.01	IBA 0.1	73.8 (4.3)	0 (0)
3b	TDZ 0.01	IBA0.5	82.5 (5.2)	32.5 (6.0)
3c	TDZ 0.01	IBA 1.0	93.8 (2.4)	42.5 (6.0)
3d	TDZ 0.1	IBA 0.1	83.8 (2.4)	52.5 (7.2)
3e	TDZ 0.1	IBA0.5	92.5 (3.2)	38.8 (9.7)
3f	TDZ 0.1	IBA 1.0	92.5 (2.5)	42.5 (9.2)
3g	TDZ 0.5	IBA 0.1	92.5 (2.5)	37.5 (6.6)
3h	TDZ 0.5	IBA0.5	95 (2.0)	47.5 (3.2)
3i	TDZ 0.5	IBA 1.0	92.5 (4.3)	53.8 (3.2)
3j	TDZ 1.0	IBA 0.1	87.5 (3.2)	36.3 (11.6)
3k	TDZ 1.0	IBA0.5	97.5 (2.5)	35 (8.4)
3l	TDZ 1.0	IBA 1.0	93.8 (2.4)	45 (3.5)
Treatment 4				
4a	TDZ 0.01	NAA 0.1	91.3 (1.3)	38.8 (7.7)
4b	TDZ 0.01	NAA 0.5	80 (2.0)	1.3 (1.3)
4c	TDZ 0.01	NAA 1.0	96.3 (2.4)	42.5 (9.5)
4d	TDZ 0.1	NAA 0.1	93.8 (1.3)	56.3 (5.5)
4e	TDZ 0.1	NAA 0.5	97.5 (2.5)	58.8 (10.9)
4f	TDZ 0.1	NAA 1.0	98.8 (1.3)	60 (5.4)
4g	TDZ 0.5	NAA 0.1	91.3 (1.3)	55 (2.0)
4h	TDZ 0.5	NAA 0.5	97.5 (1.4)	58.8 (8.5)
4i	TDZ 0.5	NAA 1.0	97.5 (1.4)	76.3 (4.3)
4j	TDZ 1.0	NAA 0.1	96.3 (2.4)	50 (4.6)
4k	TDZ 1.0	NAA 0.5	96.3 (2.4)	58.8 (7.5)
4l	TDZ 1.0	NAA 1.0	91.3 (2.4)	57.5 (6.3)
