# Supplementary figures and images for: Genetic transformation of western clover (Trifolium occidentale D. E. Coombe.) as a model for functional genomics and transgene introgression in clonal pasture legume species
Source: Plant Methods. 2013 Jul 10;9:25. doi: 10.1186/1746-4811-9-25 (PMC3716983; doi:10.1186/1746-4811-9-25)

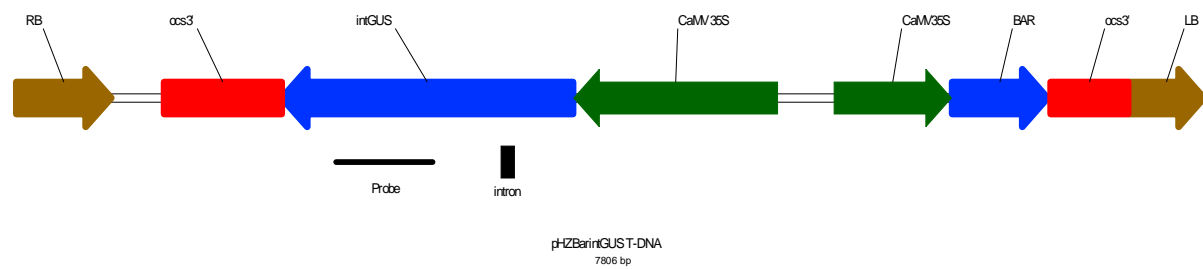

Figure S1

Supplement: Additional file 2: Figure S1 — Diagram of the T-DNA of pHZBar-intGUS. [file 1746-4811-9-25-S2.pdf]
